# Thrombosis in Systemic Lupus Erythematosus: A Review Article

**DOI:** 10.5402/2012/428269

**Published:** 2012-07-30

**Authors:** Ibrahim A. Al-Homood

**Affiliations:** College of Medicine, Imam Muhammad Ibn Saud Islamic University, P.O. Box 75227, Riyadh 11578, Saudi Arabia

## Abstract

Thrombosis is a well-known clinical entity in systemic lupus erythematosus (SLE), and it is multifactorial. The most important risk factor is the presence of antiphospholipid antibodies (APLAs). However, approximately 40% of adults with SLE who are negative for APL A are diagnosed with thrombosis, indicating the importance of other risk factors. Thus, the thrombosis risk factors should be evaluated extensively and regularly and treated aggressively in every patient with systemic lupus erythematosus.

## 1. Introduction

Systemic lupus erythematosus (SLE) is an autoimmune disease with diverse clinical manifestations that primarily affects young women. Women are affected nine times more frequently than men [1]. Of patients with SLE, 65% have disease onset between ages 16 and 55, 20% present before age 16, and 15% present after the age of 55 [2]. Estimated incidence rates in North America, South America, and Europe range from 2 to 8 per 100,000 per year [1]. Prevalence rates of SLE are estimated to be 51 per 100,000 in the United States [3], while in Saudi Arabia were estimated to be 19.28 per 100,000 population based on study done in the Al-Qaseem region [4]. Patients may be classified as having SLE if they fulfill four or more of American College of Rheumatology (ACR) classification criteria (Table 1).

## 2. Thrombosis in SLE

Patients with SLE have an increased risk for thrombosis. Arterial and/or venous thrombosis is a well-known clinical entity in SLE, with a prevalence >10%. This prevalence may even exceed 50% in high-risk patients [5]. The incidence of thrombosis in SLE patients according to two inception cohorts was 26.8 [6] and up to 51.9 per 1000 patient-years, according to the disease duration [7]. Other study reported that the incidence of thrombosis was 36.3 per 1000 patient-years [8]. In a 10-year prospective cohort study of patients with SLE, the most frequent causes of death were active SLE (26.5%), thrombosis (26.5%), and infection (25%), with thrombosis dominating the second 5-year period of followup [9]. The age at onset of thrombosis in SLE patient is lower than that of general population which is a major concern. The incidence of thrombosis increased in the first year. Possible reasons for this early higher incidence of thrombosis could be the high levels of disease activity and circulating immune complexes, cytotoxic antibodies, or a higher inflammatory state [10].

## 3. Risk Factors of Thrombosis in SLE

There are several factors that increase the thrombosis risk in SLE patients. The most important risk factors are the following.

**(1) Antiphospholipid Antibodies (APLAs)**

Antiphospholipid antibodies (APLAs) bind to plasma proteins with an affinity for phospholipids' surfaces. Most important identified antigens (ß2-glycoprotein and prothrombin) are involved in blood coagulation. Anticardiolipin antibodies (ACA) and the lupus anticoagulant (LAC) are included in classification criteria, but also anti-ß2-glycoprotein I (anti-ß2-GPI) has been confirmed to increase thrombosis risk. The prevalences of lupus anticoagulant and anticardiolipin antibodies are 28 and 42%, respectively, according to Love and Santoro review [11]. They also reported that 45% of ACA-positive patients were also LAC positive, while 59% of LAC-positive patients were also ACA positive. The risk of thrombosis in LAC/ACA patients has been examined by many authors. It appears that about one-third of all patients with LACs have had at least one thrombotic event. Among SLE patients, 42% of the LAC-positive and 40% of the ACA-positive individuals had a history of thrombosis; in contrast, the prevalence of thrombosis in LAC negative or ACA-negative SLE patients was only10–18% [11].

The antiphospholipid antibodies might be transiently positive, and to be considered significant, should be persistently positive on at least two occasions, 12 weeks apart. APLAs should be done for any patient with SLE because they considered part of ACR classification criteria for SLE not only that but also because they have associated with increased risk of thrombosis. Not all patients with APLA develop thrombosis which could be explained by different phospholipids or various binding proteins. This raised a concern about the prophylaxis which will be discussed later in this paper.

Several hypotheses have been proposed to explain the pathogenic effects of these autoantibodies and their role in the development of thrombosis. They attach to the negatively charged phospholipid surface that may induce platelet activation, interfere with coagulation inhibitors such as protein-c, inhibit antithrombin and fibrinolysis, and then initiate the formation of a thrombus. It is well established that APLAs are associated with both arterial and venous thrombosis. In Large cohort studies, the lupus anticoagulant has been shown to be a significant risk factor for myocardial infarction [12] and stroke [13].

Approximately 40% of adults with SLE who are negative for APLA are diagnosed with thrombosis [14]. Thus, the precise mechanism(s) responsible for thrombosis in these patients remains unclear and indicating the role of other factors.

**(2) Inflammation and Disease Activity**

Inflammation may affect several steps in blood coagulation: initiation, propagation, and regulation [15]. It has been shown that inflammation induces the expression of tissue factors, an important step of the initiation of coagulation [16–18]. The presence of inflammation reduces the fibrinolytic activity through upregulation of the production of plasminogen activator inhibitor (PAI) [19]. Further, the anticoagulant effect of the protein C pathway is impaired due to downregulation of thrombomodulin [20–22] and a decrease of protein S [23]. This might explain the reason of the occurrence of thrombosis early in lupus patients as compared with non-SLE controls, with odds ratio of approximately 5–10. Lupus nephritis is one of SLE clinical features that is associated with high risk for thrombosis particularly with significant proteinuria that increases the risk of deep vein thrombosis and renal vein thrombosis mainly. Different hemostatic abnormalities have been recognized in these patients, which probably account for this hypercoagulable state [24]. Systemic hypertension and hyperlipidemia are frequent in lupus nephritis and have been associated with thrombosis. Therefore, SLE itself is considered an independent risk for thrombosis.

**(3) The Other Thrombophilic Risk Factors**

Protein C, protein S, and antithrombin deficiencies are rare but carry a higher risk for venous thrombosis [25]. The protein C pathway is one of the most important anticoagulant systems. Protein C is activated on endothelial cells by thrombin bound to thrombomodulin. The activated protein C (APC) exerts its anticoagulant function by proteolytic cleavage of the procoagulant protein factors Va and VIIIa [26]. The activated protein C resistance (APC-R) is defined as a decreased anticoagulant response to the protein C pathway [27]. Hereditary APC-R caused by the factor V Leiden mutation is strongly associated with an increased risk of venous thrombosis(VTE) [28, 29]. Acquired APC-R, a phenotypic APC-R that occurs in the absence of the factor V Leiden mutation, has been reported in patients with APLA [30–34].

Male et al. [35] reported that the prevalence of acquired APC-R was significantly higher in LA-positive patients (50%) than in LA-negative patients (21%).

Factor V Leiden (FVL) is a point mutation in the factor V gene that results in the substitution of glycine for arginine at codon 506. This mutated factor V is activated normally by thrombin and, therefore, functions normally as a coagulation cofactor. However, the activated factor V is resistant to cleavage by activated protein C (APC), thus removing an important feedback inhibition of the clotting system, and conferring a risk of thrombosis, primarily venous thrombosis [36].

The G20210A prothrombin gene mutation—substitution of adenine for guanine at position 20,210 in the nucleotide sequence—is present in 2-3% of the population and in ~6% of unselected patients experiencing their first venous thrombosis [37]. The mutation does not affect the structure of prothrombin, but results in higher circulating levels of the protein [36].

Several studies had demonstrated that FVL and prothrombin G20210A mutation increased the risk of venous thrombosis (VTE), and the risk is even higher in presence of APLA. These factors alone increased the risk of VTE 20-fold, and in combination with APLA 30-fold, compared with the general population. Thrombophilic defects, in contrast with APLA, did not influence the risk of arterial thrombosis (ATE).

The plasma homocysteine level is an independent risk factor for atherosclerosis, arterial thrombosis, and possibly, venous thrombosis [38]. Elevated plasma homocysteine levels can occur as the result of deficiencies of vitamins B12 or B6 or folic acid, chronic renal failure, hypothyroidism, certain malignancies, drugs, and inherited enzyme abnormalities [36]. Prothrombotic activities may be attributable to either direct toxic effect on endothelium or indirect effects. Hyperhomocysteinemia is detected in around 15% of lupus patients. The prevalence of hyperhomocysteinemia is significantly higher in SLE patients with thrombosis. Raised levels of homocysteine are demonstrated in 27.3% of SLE patients with thrombosis compared to 16.9% in those without thrombosis [39].

**(4) The Traditional Risk Factors**

Smoking has consistently been associated with worse outcomes [40–42] and the occurrence of thrombotic events in general, and venous events in particular [43–45]. One possible mechanism by which smoking increases the risk of thrombotic events is by inducing endothelial damage, which results in the activation of the endothelium and the initiation of a cascade of events leading to thrombosis.

Hypertension, Diabetes mellitus, and dyslipidemia are not uncommon in SLE patients. These factors have been associated with thrombosis in numerous studies [46, 47].

Age has been demonstrated to be a risk factor for the occurrence of thrombotic events in SLE [48, 49]. This might be explained by the fact that older SLE patients tend to have more damage and more vascular morbidity.

**(5) Drugs and Thrombosis in SLE**

Glucocorticoids are commonly used for treatment of various manifestations of SLE. Glucocorticoids have been associated with thrombosis, probably mediated by endothelial damage and accelerated atherosclerosis [49, 50]. When administered in high doses, glucocorticoids have also been associated with abnormalities in the coagulation cascade.

Hydroxychloroquine (HCQ) is commonly prescribed antimalarial agent for SLE. It has a very reasonable safety profile and it decreases the probability of flares [51]. It has antithrombotic effect.

The antithrombotic effect is probably mediated by inhibition of platelet aggregation and adhesion, and arachidonic acid release from stimulated platelets [52]. It also decreases the thrombus size and the time of thrombus development in a dose-dependent manner [53] and inhibits the APLA-induced GPIIb/IIIa receptor expression [54]. Furthermore, it has cholesterol-lowering effects. As it reduces the risk of thrombosis as well as the disease activity, it should be started for all SLE patients.

Aspirin (ASA) inhibits the cyclooxygenase enzyme and inhibits the synthesis of thromboxane A2, a potent stimulator of platelet aggregation. Low-dose aspirin (81–100 mg) is not uncommonly used for primary thrombosis prophylaxis in high-risk patients. Among asymptomatic APLA-positive patients with SLE, primary prophylaxis with aspirin (although data are conflicting) and hydroxychloroquine appears to reduce the frequency of thrombotic events. Tektonidou et al. 2009 reported the duration of use of ASA and HCQ associated with decreased thrombosis in APLA-positive patients [55]. Tarr et al. 2007 also reported lower incidence of thrombosis in APLA-positive when given ASA prophylaxis [56]. However, Erkan et al. 2002 found that ASA was not effective in preventing thrombosis compared to placebo [57]. Therefore, there is no solid data to support the routine use of ASA for primary thromboprophylaxis. Despite that, use of low-dose aspirin has been recommended by the Expert Committee in the European League Against Rheumatism guidelines for the management of SLE [58].

## 4. Treatment Strategies of Thrombosis in SLE

Given the beneficial effects of antimalarial agents (HCQ) in patients with lupus, the use of HCQ is recommended for all patients unless it is contraindicated. Anticoagulants are used for the treatment of thrombotic events. Heparin (either intravenous or subcutaneous) and oral anticoagulation, warfarin, should be started together. Heparinization usually continues for 3–5 days to be sure therapeutic range of INR that has been achieved. If intravenous heparin is used, APTT will be used to monitor the response to establish effective heparinization. However, APTT might be prolonged by the presence of LAC. When the preheparinization APTT is prolonged, the thrombin time may be followed. The thrombin time reflects the conversion of fibrinogen to fibrin but is also sensitive to the presence of inhibitors that may be present in the plasma, for example, heparin. The reference range in general is 13–15 s. Therefore, it is sensitive to heparin, but is not prolonged by LACs and a reasonable approach would be to heparinize to a thrombin time of about 100–120 s [36]. The duration of treatment might be determined by the presence of APLA, site of thrombosis, recurrence, and presence of precipitating factors. Unprovoked thrombotic events, arterial events, or presence of APLA support prolonged anticoagulation therapy. Derksen et al. [59] found the probability of no recurrence in patients receiving oral anticoagulants at eight years to be 100%, whereas patients in whom anticoagulant drugs were stopped, the rate of recurrence was 50% at 2 years and 78% at 8 years of followup. The intensity of anticoagulation has been debated. Randomized controlled trials (Finazzi et al. 2005 [60]; Crowther et al. 2003 [61]) have suggested that INR levels between 2.0 and 3.0 are sufficient in uncomplicated antiphospholipid syndrome patients with only venous thrombotic events. However, for arterial thrombosis or recurrent events, high-intensity warfarin with INR > 3 is recommended [62]. Ruiz-Irastorza et al., 2007, [62] did a systematic review and found that the risk of recurrent events was higher in those with arterial than with venous thrombosis; therefore, it was recommended that patients with definite APS be treated with prolonged moderate-intensity warfarin in patients with first venous events and high-intensity warfarin for those with recurrent and/or arterial events. One of major complications of anticoagulation is bleeding. However, the risk of recurrent thrombotic events exceeds the risk of bleeding which is relatively low [60, 61].

## 5. Conclusions

SLE patients are at significantly high risk for thrombosis which is multifactorial. A risk-stratified approach to thrombosis risk factors is important in management of lupus. Traditional risk factors should be assessed each visit and treated rigorously. APLA should be screened in all SLE patients. Active disease particularly lupus nephritis should be treated promptly. Hydroxychloroquine has been demonstrated to reduce the disease-related morbidity and mortality and as it may reduce the thrombotic risk, it should be started for all patients unless contraindicated. The protective effect of low dose of aspirin as primary thromboprophylaxis is controversial issue. The treatment of thrombotic event is anticoagulation with target INR 2-3 for venous events and 3-4 for arterial or recurrent venous events.

## Figures and Tables

**Table 1:** American College of Rheumatology (ACR) revised classification criteria for systemic lupus erythematosus.

Criteria	Definition
Malar rash	Fixed erythema, flat or raised, over the malar eminences, tending to spare the nasolabial folds

Discoid rash	Erythematous raised patches with adherent keratotic scaling and follicular plugging; atrophic scarring occurs in older lesions

Photosensitivity	Skin rash as a result of unusual reaction to sunlight, by patient history or physician observation

Oral ulcers	Oral or nasopharyngeal ulceration, usually painless, observed by a physician

Arthritis	Nonerosive arthritis involving two or more peripheral joints, characterized by tenderness, swelling, or effusion

Serositis	(a) Pleuritis—convincing history of pleuritic pain or rub heard by a physician or evidence of pleural effusion (b) Pericarditis—documented by ECG or rub or evidence of pericardial effusion

Renal disorder	(a) Persistent proteinuria > 0.5 g/day > 3+ if quantitation is not performed (b) Cellular casts—may be red blood cell, hemoglobin, granular tubular, mixed

Neurologic disorder	(a) Seizures—in the absence of offending drugs or known metabolic derangements (e.g., uremia, acidosis, or electrolyte imbalance) (b) Psychosis—in the absence of offending drugs or known metabolic derangements (e.g., uremia, acidosis, or electrolyte imbalance)

Hematologic disorder	(a) Hemolytic anemia with reticulocytosis (b) Leukopenia—<4000/mm (c) Lymphopenia—<1500/mm (d) Thrombocytopenia—<100,000/mm in the absence of offending drugs

Immunologic disorder	(a) Anti-DNA—antibody to native DNA in abnormal titer (b) Anti-Sm—presence of antibody to Sm nuclear antigen (c) Positive finding of antiphospholipid antibodies based on (1) abnormal serum concentration of IgG or IgM anticardiolipin antibodies, (2) positive test result for lupus anticoagulant using a standard method, or (3) false-positive serologic test for syphilis known to be positive for at least 6 mo and confirmed by Treponema pallidum immobilization or fluorescent treponemal antibody absorption test

ANA	Abnormal titer of ANA by immunofluorescence or equivalent assay at any point in time and in the absence of drugs known to be associated with drug-induced lupus syndrome
